# Referral of patients with depression to mental health care by Dutch general practitioners: an observational study

**DOI:** 10.1186/1471-2296-12-41

**Published:** 2011-05-26

**Authors:** Ellen Piek, Klaas van der Meer, Brenda WJH Penninx, Peter FM Verhaak, Willem A Nolen

**Affiliations:** 1 Department of General Practice, University Medical Center Groningen, University of Groningen, Antonius Deusinglaan 1, Postbus 196, 9700 AD Groningen, The Netherlands; 2 Department of Psychiatry, University Medical Center Groningen, University of Groningen, Hanzeplein 1, Postbus 30.001, 9700 RB Groningen, The Netherlands; 3 Department of Psychiatry, EMGO Institute for Health and Care Research, VU University Medical Center, AJ Ernststraat 887, 1081 HL Amsterdam, The Netherlands; 4 Department of Psychiatry, Leiden University Medical Center, Leiden, The Netherlands; 5 Netherlands Institute for Health Services Research, Utrecht, The Netherlands

## Abstract

**Background:**

Depression is a common illness, often treated in primary care. Guidelines provide recommendations for referral to mental health care. Several studies investigated determinants of referral, none investigated guideline criteria as possible determinants.

We wanted to evaluate general practitioner's referral of depressed patients to mental health care and to what extent this is in agreement with (Dutch) guideline recommendations.

**Methods:**

We used data of primary care respondents from the Netherlands Study of Depression and Anxiety with major depressive disorder in the past year (n = 478). We excluded respondents with missing data (n = 134). Referral data was collected from electronic patient files between 1 year before and after baseline and self report at baseline and 1-year follow-up. Logistic regression was used to describe association between guideline referral criteria (e.g. perceived need for psychotherapy, suicide risk, severe/chronic depression, antidepressant therapy failure) and referral.

**Results:**

A high 58% of depressed patients were referred. Younger patients, those with suicidal tendency, chronic depression or perceived need for psychotherapy were referred more often. Patients who had used ≥2 antidepressants or with chronic depression were more often referred to secondary care. Referred respondents met on average more guideline criteria for referral. However, only 8-11% of variance was explained.

**Conclusion:**

The majority of depressed patients were referred to mental health care. General practitioners take guideline criteria into account in decision making for referral of depressed patients to mental health care. However, other factors play a part, considering the small percentage of variance explained. Further research is necessary to investigate this.

## Background

Most patients with depression are treated in primary care [1,2]. Primary care guidelines for the treatment of depression, including the Dutch guideline, recommend antidepressants and/or various forms of psychotherapy [3-7]. When psychotherapy or counselling is indicated, a general practitioner (GP) can choose to counsel the patient himself or refer the patient to another health professional [3]. In case of depression with psychotic features, a depressive episode in the course of bipolar disorder, a severe depression with social impairment or high suicide risk, or insufficient response to two or more antidepressants or other treatment, most guidelines recommend referral to secondary care [3-7]. In addition, most guidelines recommend referral for psychological interventions in certain cases, although criteria differ between guidelines [3-7]. Finally, patients with seasonal affective disorder may be referred for light therapy [3]. For the current study, we used the Dutch primary care depression guideline, which is comparable to international primary care depression guidelines.

A few studies investigating referral behaviour of GPs suggest that multiple factors play a role in whether or not a patient is referred to mental health care, including disease characteristics (diagnosis, severity of symptoms, psychiatric comorbidity, personality characteristics, somatic comorbidity), patient characteristics (age, gender, race, education, insurance policy), whether the patient presented with psychological complaints, and lastly characteristics of the GP (e.g. organization of practice, experience of the GP, and degree of urbanization) [8-15]. However, none of these studies evaluated specifically the criteria for referral as mentioned in the guidelines.

The aim of this study was to evaluate the referral practice by GPs to primary (i.e. psychologist, psychiatric nurse or social worker affiliated with the GP practice) and secondary mental health care (i.e. psychiatrist or psychotherapist in free practice, or health care professional affiliated with hospital/institute for mental health care), of patients with depression who had visited their GP for whatever reason during the past four months. First, we wanted to know how many patients with depression were referred to primary and secondary mental health care. Second, if any differences existed between non-referred and referred patients, and between patients referred to primary and secondary mental health care. Third, we wanted to know if the Dutch guideline recommendations for referral to primary and secondary mental health care corresponded with clinical practice.

We hypothesized that all criteria for referral mentioned in the Dutch guideline would independently increase the likelihood of referral. We had no hypothesis as to where most patients would be referred i.e. primary or secondary mental health care.

## Methods

This study was conducted with baseline-and 1-year follow-up data from the Netherlands Study of Depression and Anxiety (NESDA, http://www.nesda.nl), a large prospective cohort study (n = 2981) on the course of depression and anxiety disorders among respondents aged 18-65 years, recruited from the community, primary care and secondary mental health care, that started in 2004. Detailed information on the objectives and methods of NESDA were published elsewhere [16].

In The Netherlands access to secondary (mental) health care is impossible without a referral from a GP. Moreover, in The Netherlands all patients are listed with a single GP or GP practice.

At baseline an extensive interview was conducted. At 1-year follow-up all respondents filled in an elaborate questionnaire. In addition, we used data collected from the electronic patient file (EPF) of the GP for the period of one year before until one year after the baseline interview. Finally, we used data from questionnaires filled in by the GPs themselves.

### Study sample

Details on recruiting methods were published elsewhere [16]. In short a screening questionnaire was sent to a random sample of 23,750 patients from 65 GPs, who consulted their GP in the past four months irrespective of reason for consultation. The screener was returned by 10,706 persons (45%). Those screening positive were approached for a telephone interview consisting of Composite International Diagnostic Interview (CIDI) short form, which has proven diagnostic quality for screening purposes [17,18]. Those fulfilling criteria for a current disorder on the CIDI short form were invited to participate in NESDA, as was a random selection of screen-negatives (both from the written screener and telephone interview). In total 1610 persons were recruited, and underwent an extensive baseline interview, including the CIDI [19,20]. The GP was not aware of the results of the screening and interview.

From these, we included in our study only respondents with a major depressive disorder in the past year (n = 478).

We excluded respondents who did not give permission to use their EPF (n = 15) or did not fill in the 1-year follow-up questionnaire (n = 98), as we did not have full referral data on these respondents. We also excluded respondents of whom the GP had not filled in the GP questionnaire (n = 21), as we would be unable to determine the influence of GP characteristics on referral in these cases. We thus included 344 respondents in our analysis. Excluded respondents were on average younger and had a higher Inventory of Depressive Symptomatology (IDS) score at baseline.

### Indicators/guideline criteria for referral

A detailed description of all measures can be found elsewhere [16]. Demographic data (age, gender, education) were assessed during the baseline interview. Current and lifetime diagnoses of MDD based on DSM-IV were assessed with the CIDI, as well as duration of symptoms and number of previous episodes, we constructed from these data the variable chronic depression defined as >12 months with depression in the past two years. Suicidal tendency (suicidal ideations past week, suicide attempt ever) was measured with the Beck Suicide Ideation Scale [21]. Current and past use of antidepressants were based on self report, we derived from these data, which patients had stopped two or more antidepressants.

During the baseline interview number of chronic somatic diseases was recorded. The Perceived Need for Care Questionnaire (PNCQ) and Trimbos/iMTA questionnaire for Costs associated with Psychiatric Illness (Tic-P) were administered during the baseline interview to assess need for care and care received [22]. From these questionnaire we used the answers to the questions of perceived need for psychotherapy and perceived need for any other treatment.

Finally, we used several GP and practice characteristics (years of experience as a GP, self-reported interest in depression, presence of a social worker, social psychiatric nurse or psychologist in the GP practice), derived from the GP questionnaires.

Table 1 shows a summary of indicators/guideline criteria used.

**Table 1:** Indicators/guideline criteria for referral

Guideline criteria for referral	Other patient characteristics	GP/practice characteristics
Stopped two or more antidepressants	Age	Years of experience as a GP
Perceived need for (more) psychotherapy/counselling	Gender	Special interest in depression
Perceived need for more or other treatment other than psychotherapy/counselling	Presence of chronic somatic diseases	Presence of mental health professional in GP practice
More than 12 months with depression in past two years	Comorbid anxiety disorder past year	
Suicidal ideations past week or suicide attempt ever		

### Target variables

We constructed the variable "referral", indicating whether or not referral had taken place. Referral was considered present when a letter to or from a mental health professional was present in the EPF or when the respondent reported contact with a mental health professional in the past 6 months at baseline as measured with the PNCQ and Tic-P and in the past year with the Tic-P at 1-year follow-up.

We also created a variable indicating whether referral had been to primary (i.e. a psychologist, psychiatric nurse or social worker affiliated with the GP practice) or secondary mental health care (i.e. a psychiatrist or psychotherapist in free practice, or any health care professional affiliated with a hospital or institute for mental health care). Exact content of treatment by each mental health professional could not be determined.

### Ethics

The study protocol of NESDA was approved centrally by the Ethical Review Board of the VU University Medical Center and subsequently by local review boards of each participating center. After full verbal and written information about the study, written informed consent was obtained from all participants at the start of baseline assessment. A full ethics statement of NESDA is found elsewhere.

### Statistical analysis

Results were presented with descriptive statistics: qualitative variables with absolute and relative frequencies, quantitative variables with means and standard deviation. Differences between two groups were tested with Chi square test (qualitative variables) and Student's t test (quantitative variables). Logistic regression was used to estimate association of referral with observed parameters. Differences were considered significant when the p-value was < 0.05. All variables with a bivariate correlation with p-value ≤0,150 were eligible for multivariate analysis. We excluded from these variables one of each pair with a mutual correlation > 0.7 and dichotomous variables with less than 5.0% of respondents in one of the groups. For this multivariate analysis, logistic regression was used, with the dichotomous variable "referral" as dependent variable. We used a manual stepwise backward method to remove non-significant variables.

All statistical analyses were performed with the "Statistical Package for the Social Sciences" version 16.0 (SPSS Inc., Chicago).

## Results

Of the 344 respondents with MDD, 241 were female and 103 were male with an average age of 45.5 years (SD 11.7). 199 (57.8%) were referred to mental health care and 145 (42.2%) were not. Of the 199 referred, 93 (46.7%) were referred to primary mental health care and 106 (53.3%) to secondary mental health care.

We compared referred and non-referred respondents on the guideline criteria and patient and GP/practice characteristics (table 2). Suicidal tendency, chronic depression (≥12 months with depression in past two years) and perceived need for psychotherapy were more often present in the referred group, these patients were on average younger. None of the GP or practice characteristics were significantly different between groups.

**Table 2:** Differences between respondents with and without referral to mental health care

	No referral	Referred	Total	OR (95% CI)	p-value
N (%)	145 (42.2%)	199 (57.8%)	344(100%)	N/A	N/A
Stopped two or more antidepressants	10 (6.9%)	23 (11.6%)	33 (9.6%)	1.76 (0.81 - 3.83)	0.147
**Perceived need for (more) psychotherapy/counselling **	**59 (40.7%) **	**118 (59.3%) **	**177 (51.5%) **	**2.12 (1.37 - 3.28) **	**0.001 **
Perceived need for (more) treatment other than psychotherapy/counselling	81 (55.9%)	110 (55.3%)	191 (55.5%)	0.98 (0.64 - 1.50)	0.914
**More than 12 months with depression in past two years **	**31 (21.4%) **	**63 (31.7%) **	**94 (27.3%) **	**1.70 (1.04 - 2.80) **	**0.035 **
**Suicidal ideations past week or suicide attempt ever **	**35 (24.5%) **	**79 (39.7%) **	**114 (33.3%) **	**2.03 (1.26 - 3.27) **	**0.003 **
Age*	**47.10 (11.93) **	**44.32 (11.48) **	**45.49 (11.74) **	**N/A **	**0.031 **
Gender, male	38 (26.2%)	65 (32.7%)	103 (29.9%)	0.73 (0.46 - 1.18)	0.197
Chronic diseases	102 (70.3%)	127 (63.8%)	229 (66.6%)	0.74 (0.47 - 1.18)	0.205
Comorbid anxiety disorder past year	89 (61.4%)	128 (64.3%)	217 (63.1%)	1.13 (0.73 - 1.77)	0.577
Years of experience as a GP*	18.21 (10.49)	19.89 (9.88)	19.18 (10.16)	N/A	0.133
Special interest in depression	40 (29.9%)	52 (27.8%)	92 (28.7%)	0.91 (0.56 - 1.48)	0.690
Presence of mental health professional in GP practice	101 (69.7%)	147 (73.9%)	248 (72.1%)	1.23 (0.77 - 1.98)	0.390

N = absolute number of respondents; (%) = percentage within variable referral; OR = Odds Ratio; (95% CI) = 95% Confidence Interval for Odds ratio, p-value is from chi-square test

* Numbers are mean (standard deviation), p-value is from independent samples t-test

Next, we compared the groups referred to primary and to secondary mental health care (table 3). Having stopped two or more antidepressants and chronic depression, were more common in respondents referred to secondary mental health care.

**Table 3:** Differences between respondents referred to primary and secondary mental health care

	Primary mental health care	Secondary mental health care	Total	OR (95% CI)	p-value
N (%)	97 (46.2%)	113 (53.8%)	210 (100%)	N/A	N/A
**Stopped two or more antidepressants **	**5 (5.4%) **	**18 (17.0%) **	**23 (11.6%) **	**3.60 (1.28-10.12) **	**0.011 **
Perceived need for (more) psychotherapy/counselling	54 (58.1%)	64 (60.4%)	118 (59.3%)	1.10 (0.62 - 1.94)	0.740
Perceived need for (more) treatment other than psychotherapy/counselling	47 (50.5%)	63 (59.4%)	110 (55.3%)	1.43 (0.82 - 2.52)	0.208
**More than 12 months with depression in past two years **	**20 (21.5%) **	**43 (40.6%) **	**63 (31.7%) **	**2.49 (1.33 - 4.67) **	**0.004 **
Suicidal ideations past week or suicide attempt ever	32 (34.4%)	47 (44.3%)	79 (39.7%)	1.52 (0.86 - 2.70)	0.153
Age*	43.42 (11.64)	45.10 (11.34)	44.32 (11.48)	N/A	0.304
Gender, male	29 (31.2%)	36 (34.0%)	65 (32.7%)	0.88 (0.49 - 1.60)	0.677
Chronic diseases	57 (61.3%)	70 (66.0%)	127 (63.8%)	1.23 (0.69 - 2.19)	0.487
Comorbid anxiety disorder past year	54 (58.1%)	74 (69.8%)	128 (64.3%)	1.67 (0.93 - 3.00)	0.084
Years of experience as a GP*	20.97 (9.33)	18.94 (10.28)	19.89 (9.88)	N/A	0.147
Special interest in depression	23 (26.4%)	29 (29.0%)	52 (27.8%)	1.14 (0.60 - 2.16)	0.696
Presence of mental health professional in GP practice	65 (69.9%)	82 (77.4%)	147 (73.9%)	1.47 (0.78 - 2.78)	0.232

N = absolute number of respondents; (%) = percentage within variable referral; OR = Odds Ratio; (95% CI) = 95% Confidence Interval for Odds ratio, p-value is from chi-square test

* Numbers are mean (standard deviation), p-value is from independent samples t-test

Subsequently, we tested all variables from table 1 bivariately against the dependent variable "referral". Age and gender were tested, in order to control for them in the model, if they where significant. All variables with a bivariate p-value ≤0.150 were entered into the model, after step-wise backward deletion, only need for (more) psychotherapy and suicidality remained significant, when controlled for age. This model explained eight to eleven percent of variance (table 4).

**Table 4:** Results of multivariate binary logistic regression analysis with referral as dependent variable

	Odds ratio*	95% Confidence interval	P-value
Age	0.974	0.955 - 0.994	**0.010 **
Stopped two or more antidepressants	1.634	0.706 - 3.784	0.252
Perceived need for (more) psychotherapy/counselling	1.865	1.187 - 2.930	**0.007 **
More than 12 months with depression in past two years	1.713	0.995 - 2.948	0.052
Suicidal ideations past week or suicide attempt ever	1.810	1.098 - 2.985	**0.020 **

Odds ratio for referral in case criterion present, except for criterion age, where odds ratio represents odds for referral with each year increase in age.

**Figure 1:**
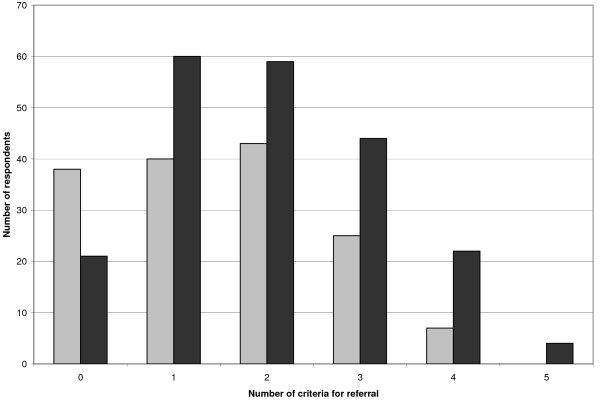
**Number of respondents (not) referred in relation to number of criteria for referral**. □ Not referred. ■ Referred.

Finally, we tested whether the number of criteria present would predict the chance of referral. Indeed, referred respondents met significantly more criteria (median 2.00) than non-referred respondents (median 1.00), p = 0.000. Respondents with one or more criteria had an odds ratio of referral compared to respondents without any criteria of 2.70 (95% CI for odds ratio 1.49 - 4.87). Relation between referral and number of criteria is graphically depicted in Figure 1.

## Discussion

### Strengths and limitations of the study

Our study has several strong points. First, as a screening method was used to recruit participants and all respondents were interviewed by independent interviewers, GPs were unaware of the psychiatric diagnosis. They could only rely on their own judgement in their treatment decisions, including referral. Second, the use of a structured interview (CIDI) for diagnosis. Third, with the extensive interview of NESDA, almost all relevant criteria for referral could be assessed.

There are also limitations. First, the specific Dutch situation where GPs are gatekeepers to mental health care, rendering it unclear if these results can be generalized to countries with other referral systems. Second, we were unable to examine certain criteria presented in the guideline, such as comorbid personality disorders (also a guideline criterion for referral), as these were not assessed in NESDA. Third, missing data on diagnoses in 30% of the GP EPFs, rendering it impossible to clarify the influence of "recognition" on referral practice. Fourth, we were unable to investigate the influence of symptom severity on referral, as referral was initiated at a different point in time for each participant, while symptom severity was measured at baseline and 1-year follow-up only. The symptom severity at time of referral could be very different from the symptom severity at any of these set points in time.

### Comparison with existing literature

Referral rate in our study (almost 58% of patients with MDD in the past year) was high compared to the previous studies. Kendrick et al. reported an overall percentage of 22.8%, including patients with minimal or mild depressive symptoms according to either the 9-item Patient Health Questionnaire or the depression subscale of the Hospital Anxiety and Depression Scale [23]. In the study by Wang et al. 26% of patients with a "mental health visit" and 25% with any visit to their GP were referred to a psychologist or psychiatrist [14]. Finally, Grembowski et al. found that 23% of patients with depressive symptoms were referred, while 38% had contact with a mental health specialist, as this was also possible without referral [24]. These differences are probably a result of different populations of patients and methods for diagnosing depression: GP diagnosis, questionnaires such as Hospital Anxiety and Depression Scale or structured interviews like the CIDI, and different definitions of referral and mental health care. Kendrick et al. included patients with minimal or mild depressive symptoms; Grembowski et al. patients with depressive symptoms, from both groups at least some patients would probably not fulfil criteria for MDD as used in our study [23,24]; Wang et al. only considered referrals to psychiatrists or psychologists, while we also investigated social workers, social psychiatric nurses, psychotherapists and professionals in institutes for mental health care [14]; and lastly, the study by Grembowski et al. was performed in the United States where a referral from a GP is not required to see a specialist [24].

Although none of the studies investigated all of the determinants we did, several of our determinants were investigated by others. Younger age was associated with more referral in our study, and in the study of Wang et al. and Grembowski et al. [14,24] The incidence of comorbid anxiety disorder did not differ between groups, in line with the study by Simon et al. [25] Referral rates for males and females were the same in our study in concordance with the studies of Miller et al. and Simon et al., but in contrast to the studies by Grembowski et al. and Kendrick et al. [23-26] Chronic somatic disease did not significantly differ between referred and non-referred patients in our population either, in contrast to the study of Kendrick et al. and Miller et al. [23,26]

### Implications for future research and clinical practice

Our study shows that Dutch GPs use guideline criteria in their decision to refer depressed patients to mental health care. However, the small percentage of explained variance by the guideline criteria for referral in our multivariate model suggests that there is room for further improvement in clinical practice. If GPs would adhere strictly to the guidelines, a higher percentage would have been expected. At the same time, the small percentage of explained variance opens a door toward future research: it shows that also other factors (including patient factors) play a part in the decision making process. Future research should focus on investigating these factors. If we better understand why patients are referred (or not referred), courses on recognition and treatment of depression could educate GPs in these areas so that they might be able to take even better care of their patients. Also, while recommendations towards the need for secondary mental health care in depression are quite clear in most guidelines, the indications and possibilities of primary mental health care are less so. This could also be an interesting field of research.

## Conclusions

The majority (210/363; 57.9%) of patients with depression in primary care was referred to a mental health professional while GPs seem to apply the guideline criteria when making decisions about referral. Our hypothesis that all guideline criteria independently increased referral chance, was rejected, still suicide risk, chronic depression and patient preference for psychotherapy rendered referral more likely. Failure of treatment (chronic depression and/or stopped treatment with ≥2 antidepressants) led more often to referral to secondary mental health care.

## Competing interests

E Piek: None declared

K van der Meer: None declared

BWJH Penninx: None declared.

PFM Verhaak: None declared

WA Nolen: received grants form the Netherlands Organisation for Health Research and Development, the European Union, the Stanley Medical Research Institute, Astra Zeneca, Eli Lilly, GlaxoSmithKline and Wyeth, received honoraria and speaker's fees from Astra Zeneca, Eli Lilly, Pfizer, Servier and Wyeth and participated in advisory boards of Astra Zeneca, Cyberonics, Pfizer and Servier

## Authors' contributions

EP, KM and WAN designed the study, BWJHP and PFMV critically revised the design. EP did the interpretation of the data and the writing of the manuscript; KM, PFMV and WAN contributed in the interpretation of the data and in writing/supervising of this manuscript. BWJHP critically revised the manuscript. All authors read and approved the final manuscript.

BWJH Penninx is guarantor of the study.

## Author's information

EP is a GP and PhD-student conducting research on major depressive disorder in general practice.

KM is a emeritus professor in general practice, interested in psychiatric disorders in general practice.

BWJHP is professor of psychiatric epidemiology and PI of NESDA.

PFMV is extraordinary professor of mental health care.

WAN is a professor in psychiatry, with special interest in affective disorders.

## Pre-publication history

The pre-publication history for this paper can be accessed here:

http://www.biomedcentral.com/1471-2296/12/41/prepub
